# Mr. Evans’ New Amalgam

**Published:** 1850-01

**Authors:** Thos. W. Evans

**Affiliations:** 15 Rue de la Paix, Paris


### MR. EVANS' NEW AMALGAM.

In No. 4, Vol. 2, we published a letter from Thomas W. Evans, Dentist, of Paris, France, to the Editor of the London Lancet, in relation to a new amalgam for filling teeth.

We see from a more recent letter to Prof. C. A. Harris, that his further experiments with this article have not been so satisfactory. From an examination we recently made of two fillings of this article, we were led to regard it as no better, if not inferior to the old and oft repudiated article. They had not hardened so well, perhaps the material may not have been as well prepared as it might have been—of this we cannot judge; we only know that it had been in the tooth three or four days, and was still *very soft.*

We give, however, this last letter, that Mr. Evans may place himself right before the profession.—[J. T. Cin. Ed.

Paris, Sept. 20th, 1849.

Dr. C. A. Harris—*Dear Sir.*—In compliance with a promise given to my professional brethren, I should like to have inserted in the "American Journal of Dental Science," if space permits, my present views in regard to the preparation of tin and cadmium, an account of which I published in the London Lancet and London Medical Gazette. It appears to have been inferred from my remarks in these journals, that I am an advocate of the practice of filling teeth with amalgams. This is an honor to which I have not the slightest claim whatever—and I must therefore leave it entirely to the enjoyment of those who are justly entitled to it. The fact is well known to my friends on this side of the Atlantic, as well as in America, that

I have always been strongly opposed to the use of any other material for filling teeth than gold; and in my own practice, I have never made use of any other. My predominant opinion has always been decidedly in favor of gold, notwithstanding I have, in common with others, experimented, with a view, on my part, of testing the merits of substances designed to be used in a plastic state. From my experiments with the preparation alluded to, I discovered it possessed qualities that other amalgams did not. Its preserves its color better than the other mercurial preparations I am acquainted with. It also has the advantage of becoming a tough, ductile substance, susceptible of being cut or burnished like a piece of tin. In addition, the cadmium seems to completely absorb the mercury in the process of crystallization. From these circumstances, it was thought to possess important advantages over any of the substances hitherto employed.

There is, in my opinion, a very great objection to all the combined metals, arising from the fact that they are all promoters of galvanic action. This objection is a very serious one in a substance employed for the purpose of filling teeth. Upon removing some of this filling, which appeared perfectly tight, and which was unchanged in its color upon the exterior surface, I have found that beneath the metal a deep yellow hue had made its appearance, penetrating for some distance into the bone of the tooth. This phenomenon does not present itself till after the lapse of considerable time, and appears much more striking in some cases than in others. Whether this effect is to be ascribed to galvanic agency, yet remains to be determined. Mr. Faraday observes, (see Turner's Chemistry, 6th Am. edition, p. 399,) that an alloy of steel with one-hundredth part of its weight of platina, dissolves with effervescence in diluted sulphuric acid, so weak as scarcely to act on common steel; a fact which he accounts for by ascribing it to the steel being rendered positive by the presence of the platina.

I have watched the effects produced by the application of this preparation, and the result of my observation has been such as I have stated above. I am now engaged in investigating the subject of the different amalgams that have been in use, in reference to their influence upon the general health; and I hope soon to be able to give the opinion of some of the highest medical authorities in Europe, upon a question which has elicited so much discussion, and which, in reality, is one of the greatest importance.

In giving publicity to this preparation, the object was not simply to make known the result of my experiments, but also to invite the attention of the profession to a subject so peculiarly important as that of discovering a material to be used *in those cases in which the circumstances might seem to indicate the expediency of employing a soft filling*, and to take the place of the compositions deemed objectionable, already in use. Experience, the great test in such matters, has furnished a result not so favorable as was anticipated by many members of the profession, who had expressed their decided approbation of this preparation, and who seemed perfectly confident that it was destined to fill a great desideratum which had so long been felt.

Whatever may be the hopes of others regarding this preparation, I have never considered it a substitute for gold. I have regarded it as a mere expedient, to be resorted to only in *peculiar* cases.

In regard to its influence upon the general health, in the opinion of those whose authority is entitled to weight, it is not injurious in this respect. *I cannot, however, refrain from stating it as my deliberate opinion, that all operations in which amalgams are employed, are merely temporary in their nature, and that any tooth that can be filled in a* PROPER *manner with gold, can be effectually and permanently saved only by this means.*

This is the opinion I have always entertained, and I adhere to it at the present moment, with *undiminished* confidence.

Yours, respectfully,

THOS. W. EVANS.

15 Rue de la Paix, Paris.

